# Performance Enhancement of an Ultrasonic Power Transfer System Through a Tightly Coupled Solid Media Using a KLM Model

**DOI:** 10.3390/mi11040355

**Published:** 2020-03-30

**Authors:** Bibhu Kar, Ulrike Wallrabe

**Affiliations:** Laboratory for Microactuators, IMTEK - Department of Microsystems Engineering, University of Freiburg, Georges-Koehler-Allee 102, Room 02-087, 79110 Freiburg, Germany; bibhu.kar@imtek.uni-freiburg.de

**Keywords:** piezoelectric transducer, ultrasonic power transfer, through metal wall power transfer, KLM model, contactless energy transfer

## Abstract

Contactless ultrasonic power transmission (UPT) through a metal barrier has become an exciting field of research, as metal barriers prevent the use of electromagnetic wireless power transfer due to Faraday shielding effects. In this paper, we demonstrate power transfer through a metal wall with the use of ultrasonic waves generated from a piezoelectric transducer. Accurate characterization and modeling of the transducer and investigation of the influence of the acoustic properties of the transmitting medium are instrumental for the performance prediction and optimal design of an ultrasonic power link. In this work, we applied the KLM model for the emitting and receiving transducers, with respect to the transmitting medium and model for both the emission and reception function. A practical UPT system was built by mechanically coupling and co-axially aligning two composite transducers on opposite sides of a transmitting medium wall. The optimal transmission performance of the ultrasonic power link through thickness-stretch vibrations of the wall together with two piezoelectric transducers working in TE mode was determined. Eventually, the operating frequency and ohmic loading condition for maximum power transmission were obtained for two different media, aluminium and polyoxymethylene (POM), with contrasting specific acoustic impedances. The results showed that the measured optimal electric loads and operating frequency for maximum power transfer agreed well with the theoretical predictions.

## 1. Introduction

Many sensing applications rely on electronics that are deeply embedded within metal structures, for example for condition monitoring, or behind a wall in metallic tubes and tanks to measure gas or fluid supply and chemical processing. Traditionally, to power and communicate with sensors located in hermetic metallic structures, physical penetration of the structures by feedthrough wires is applied. This can provide a significant disadvantage, as each feed-though is a potential source of leakage and failure. To maintain the structural integrity of the system, wireless energy transfer (WPT) through metal walls becomes mandatory. The well-known and established electromagnetic principles, either by inductive coupling or electromagnetic waves, are inhibited here due to the strong Faraday shielding effect presented by the metal barriers [1]. A relatively new alternative to wireless electromagnetic power transfer is acoustic/ultrasonic energy transfer. Ultrasonic waves generated by a piezoelectric transducer can transmit vibrational energy through a metal barrier, which in turn is received by a secondary piezoelectric receiver on the other side of it [2,3,4]. Therefore, ultrasonic power transfer (UPT) is discussed to be the most workable solution for WPT through a metal barrier [5], and reasonable efficiency and power levels can be obtained [6,7].

**Figure 1:**
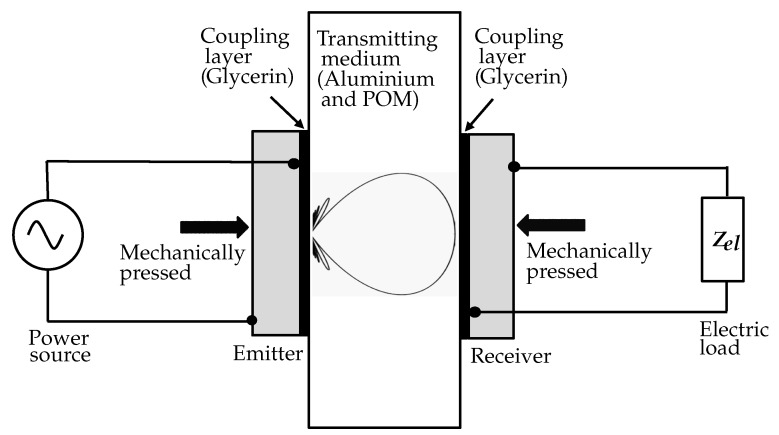
Simplified diagram of an acoustic power link, consisting of an emitting unit connected to a power source, a transmitting medium, and a receiving unit.

We study the system illustrated in Figure 1, in which a planar aluminium plate represents the metal wall of a sealed container. An acoustic-electric channel is formed by coaxially aligning and acoustically coupling a pair of piezoelectric transducers to both sides of the plate. Glycerin is used to couple the transducers into the medium, which is evidently used in ultrasonic nondestructive testing [8]. In [9], the influence of different coupling methods between the transducer and the metal wall on power transfer efficiency was investigated. The coupling with a mechanical clamp provides the highest efficiency, as we can achieve the thinnest possible coupling layer, and it avoids possible losses due to an intermediate epoxy layer. This method also ensures reusing the transducers for several experiments, e.g., to investigate the reproducibility of the measurements. As shown in Figure 1, the transducers were mechanically pressed to the metal wall using Neoprene rubber with low specific acoustic impedance on the backside to minimize the emission losses.

For the approach illustrated in Figure 1, the energy transmission depends heavily on the properties of the piezoceramic material [10], the acoustic impedance of the media [11], and the media attenuation [12], as well as the transmission frequency [13] and the attached electric loads [14]. In [14], the zero reflection and power maximization condition for a similar power link were compared to each other with water as the transmission medium. In this work, we evaluated the attached electric loads at the receiver for power maximization with aluminium as the transmitting medium using the KLM model of the receiver and subsequently validated our predictions by comparing our theoretical predictions to the measurements.

The acoustic impedance of the medium is an essential parameter concerning the transfer of acoustic energy between two media. Piezoelectric ceramics usually have a larger specific acoustic impedance than the usual acoustic loads (i.e., water, tissue, and most metal). As a consequence, a significant amount of ultrasonic energy is reflected back at the piezoelectric transducer and the medium interface, and only the remaining part enters the target medium. With aluminium as the transmitting medium, we profit from a much higher acoustic impedance, reducing the impedance mismatch between the transducers and the transmitting medium. To study the influence of the acoustic impedance of the medium on power transfer characteristics a bit more in detail, we also conduct our experiments with polyoxymethylene (POM) as an alternative transmitting medium with lower impedance.

## 2. KLM Model for Acoustic Power Link

To determine the electromechanical properties of the transducer in terms of the conversion of electric energy to mechanical energy and vice versa, many one-dimensional electric models have been proposed in the literature. Two of the most commonly used equivalent circuit models are the Mason [15] and the KLM model [16]. In [17], Sherrit et al. compared the Mason model to the KLM model and discussed the primary limitations of both models. These equivalent circuit models are instrumental in predicting the response of a piezoelectric transducer; however, since they are one-dimensional, only a one-dimensional pressure field can be investigated.

Compared to the KLM model, the Mason model uses a virtual negative capacitance, whereas the KLM model clearly distinguishes between the acoustic and electric parts of the transducers at a low computational cost [18]. Henceforth, in this work, we used the KLM model to describe an equivalent circuit that represents the ultrasonic piezoelectric transducer.

In [19], Yang et al. investigated different modeling techniques for ultrasonic energy transfer through a metal wall, including equivalent circuit models, and compared the experimental results with Mason’s equivalent circuit model and Leach’s equivalent circuit model [20]. In this work, we used the KLM model to evaluate our emitter and receiver separately, and wave bursts were used to power the emitter to avoid standing waves in the medium. We kept the pulse repetition interval six times larger than the pulse length so that the predecessor had faded away before generating a new burst. This approach made our transmitting medium infinite, and the emitter did not influence the receiver and vice versa. From an acoustic point of view, we could achieve distance-independent energy transmission by avoiding the standing wave between the emitter and receiver [14].

**Figure 2:**
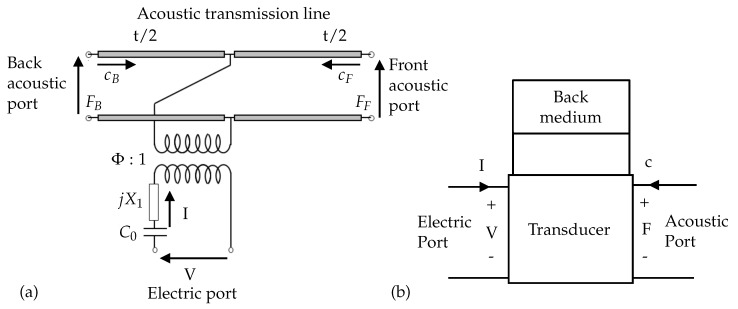
(**a**) KLM model of the transducer. (**b**) Two-port representation of a three-port KLM model of the transducer, when the back acoustic port is closed with an impedance.

As shown in Figure 2a, the KLM model for each transducer has one electric port and two acoustic ports connected through the use of an electromechanical transformer [21].

From Figure 2a, we can see that the electric port of the model consisted of a clamped capacitance $C_{0}$ and a second reactive term $jX_{1}$. The mechanical port of the KLM circuit is equivalent to a lossy acoustical transmission line, and ϕ is the transformer ratio of electric voltage to mechanical force. Additionally, *t* is the thickness of the transducer. The circuit parameters of the model were explained in [16]. The KLM model offered great flexibility to introduce different acoustic boundary conditions, and it allowed us to straightforwardly introduce different components such as electrodes, the coupling layer, and parasitic loads as concatenating matrices [22].

We closed the back mechanical port with an absorbing material (ideally air) of characteristic impedance $Z_{B}$, and the transducer could be further simplified to a two-port linear and reciprocal network, as shown in Figure 2b [18]. As a consequence, we could explain the system represented with a transfer matrix formalism using ABCD parameters:

(1) $$\left[\begin{array}{c} F\\ c \end{array}\right]=\left[\begin{array}{cc} A & B\\ C & D \end{array}\right]\left[\begin{array}{c} V\\ I \end{array}\right].$$

Here, *V* is the voltage across the electrodes of the transducer; *I* is current through the electrodes of the transducer element; *F* is the force of the ultrasound waves on the front face; and *c* is the particle velocity at the front face.

As shown in Equation (1), the KLM equivalent circuit could be reduced to a single matrix by concatenating the matrices of all the components. Using the KLM model, we could obtain any relation between the four remaining unknowns *V*, *I*, *F*, and *c* from the transfer matrix of the circuit theory, relating the parameters from the electric port to the acoustic port and vice versa. We used this procedure for the performance analysis of the transducer either as an emitter or as a receiver.

To maximize the energy transmission efficiency across the ultrasonic power link, we had to maximize the power available for the device connected to the receiver. In [23], the condition of “zero reflection at the receiver” was investigated. Thereby, the acoustic impedance of the receiver $Z_{rec}$ was evaluated with consideration of the backing and attached electric load. By selecting the proper electric load, $Z_{rec}$ could be matched to the acoustic impedance of the medium $Z_{med}$ at any operating frequency. However, an experimental verification of the zero reflection condition was difficult due to acoustic attenuation of the transmitting medium. Furthermore, to incorporate backward data transmission, zero reflection at the receiver was undesirable.

In this paper, we considered a second approach as explained in [13,24], which defined the electric load that maximized the power dissipation at the attached electric load in the receiver. *F* in Equation (1) at the front face of the receiver was a combination of forward and backward traveling waves due to acoustic impedance mismatch. Unlike the zero reflection condition, where the backward traveling wave was nullified so that all the incoming energy was absorbed by the receiver, in this approach, we maximized the power received by the attached electric load with respect to the forward traveling wave (without considering the reflected energy) and numerically evaluated the optimal electric load for power maximization using the KLM model.

## 3. Transducer Characterization

Pure PZT transducers present a high acoustic impedance of around 35 MRayl compared to aluminium (13.6 MRayl) and POM (3.4 MRayl). If an acoustic matching layer is not used, this leads to high reflection losses at the transducer and medium interface [25]. Also, PZT plates can be used either in a radial [26] or a thickness mode [23] and to realize a pure thickness resonance without any spurious modes, PZT plates require a minimum aspect ratio (diameter/thickness) of 20 [27]. To eradicate these limitations of pure PZT transducers, we used 1–3 composite piezoelectric circular plate transducers with thickness *t* (≈2 mm) and diameter *D* (≈16 mm), resonating in thickness extension (TE) mode and also polarized in the thickness direction.

The transducers were pressed against the aluminium or POM wall from their backside using Neoprene pads to minimize emission losses. Here, the piezoelectric transducer had a specific acoustic impedance $Z_{0}$ = 25.6 MRayl; the Neoprene polymer backing $Z_{B}$ = 1.55 MRayl; aluminium and POM as the transmitting medium had an impedance of $Z_{L}$ = 13.6 MRayl and 3.4 MRayl, respectively.

**Figure 3:**
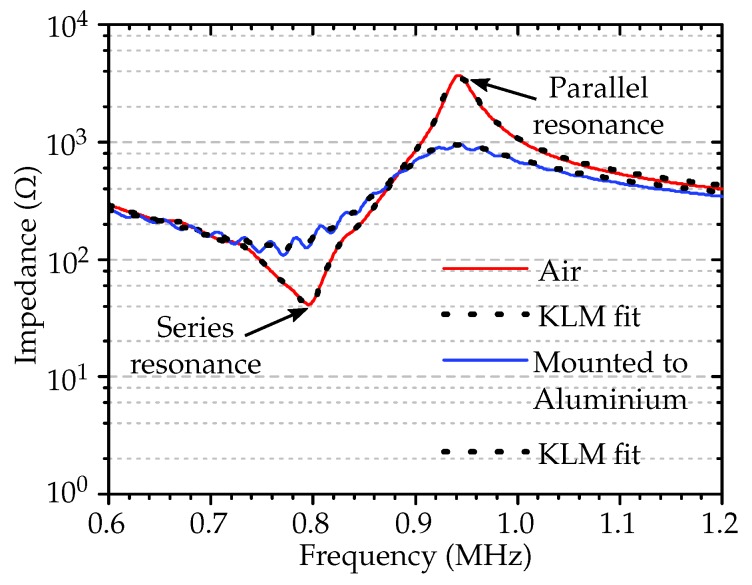
Measured electrical impedance (lines) and the KLM model fit (dots) of the transducer in air and after being mounted to the aluminium.

In order to take into account the boundary condition of the mounting structure, we measured the electric impedance of the transducer in air before mounting and after mounting it to the medium using the impedance analyzer. The measured electrical impedance data were imported to find the series resonance and parallel resonance frequencies near TE mode, as shown in Figure 3.

The desired material parameters were calculated as explained in [28]. To account for losses, the open circuit stiffness $C_{33}^{D}$, clamped permittivity $\varepsilon_{33}^{D}$, and coupling factor $k_{t}$ were treated as complex variables. The complex material parameters were assumed not to depend on the frequency, and attention must be taken in consideration of the sign of the imaginary parts of complex parameters for accounting for the losses in the piezoceramic material (Table 1). The mounting affected the electrical impedance, as shown in Figure 3, and we extracted the piezoelectric material properties mentioned in Table 1 by fitting the measured impedance to the KLM model.

## 4. Modeling of the Ultrasonic Transducer as the Emitter and Receiver

We analyzed the transducer performances separately for the emitter and receiver by means of the transfer functions. The transfer function of the transducers were obtained using the KLM model.

**Figure 4:**
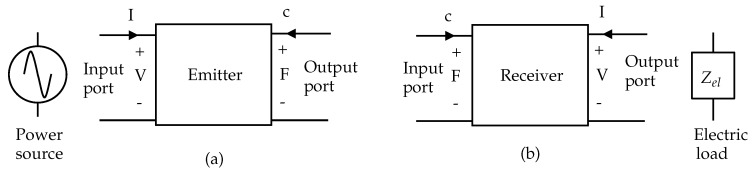
(**a**) Two-port block diagram of the emitting transducer. (**b**) Two-port block diagram of the receiving transducer.

Figure 4 shows the simple two-port linear representation of the ultrasonic transducer as the emitter and as the receiver. The emission transfer $H_{E}\left(p\right)$ as shown in Equation (2), was obtained through the KLM model. The KLM model was transformed into cascaded matrices from the electric port to the acoustic port [29]. Similarly, we computed the reception transfer function $H_{R}\left(p\right)$ as shown in Equation (3) from the acoustic port to the electric port:

(2) $$H_{E}\left(p\right)=\frac{F_{E}}{V_{ex}}$$

and:

(3) $$H_{R}\left(p\right)=\frac{V_{R}}{F_{R}}.$$

Here, $F_{E}$ is the force on the front face of the emitter; $V_{ex}$ is the excited voltage across the electrodes of the emitter; $V_{R}$ is the voltage induced on electrodes of the receiver; and $F_{R}$ is the force of the ultrasound waves on the front face of the receiver.

**Figure 5:**
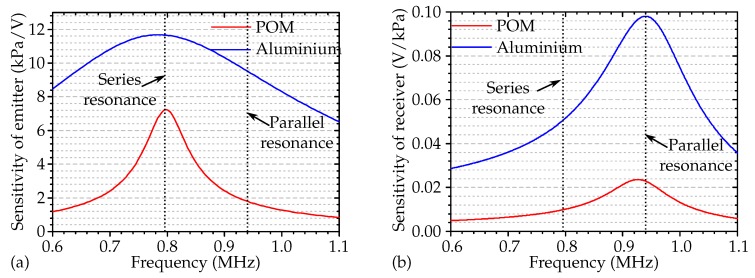
(**a**) Emission transfer function and (**b**) receiving transfer function of the transducer, for aluminium and POM as the transmitting media.

Figure 5a shows the simulated frequency response of the ultrasonic emission of a piezoelectric transducer and illustrates the role of the acoustic impedance of the involved materials. The amount of reflected or transmitted energy at the transducer and transmitting medium interface were related to the reflection $R_{o}^{L}$ and transmission $T_{o}^{L}$ coefficients, which were defined with the acoustic impedances of the two media, as shown in Equations (4) and (5) [30]:

(4) $$R_{o}^{L}=\frac{Z_{L}-Z_{0}}{Z_{L}+Z_{0}}$$

and:

(5) $$T_{o}^{L}=\frac{2Z_{L}}{Z_{L}+Z_{0}}.$$

In addition, the acoustic impedance mismatch determined the bandwidth of the transducer. All these effects are obvious from the simulation results in Figure 5a.

With this set of variables, the simulated acoustic pressure emitted into aluminium was much higher and showed broadband characteristics compared to POM due to the low acoustic impedance mismatch, and maximum output occurred at the fundamental mechanical resonance frequency $f_{0}$. However, in Figure 5b, the maximal receiving sensitivity, once the wave had traveled through the media, was shifted towards a higher frequency range (parallel resonance). The simulations thus proved that with an emitter operating at resonance frequency and transmitting ultrasonic waves into the transmitting media, but a receiver with similar characteristics did not necessarily operate at the same frequency with maximal sensitivity.

## 5. Experiments and Results

We used the previously mentioned 1–3 composite transducer with an 80% filling factor, a diameter of 16 mm, and a thickness of 2 mm as emitter coupled to one side of the medium wall and a similar receiver on the other side of the wall. The exact resonant frequencies of the transducers were measured using an Agilent 4395A impedance analyzer (Agilent Technologies, Santa Clara, CA, USA) before and after bonding to the medium plate, and the results are presented in Figure 3. We evaluated the resonance behavior and characterized the transducers operating in TE mode. We used a 20 mm thick aluminium and a 40 mm thick POM as the transmitting media, outside the near-field of the emitter.

**Figure 6:**
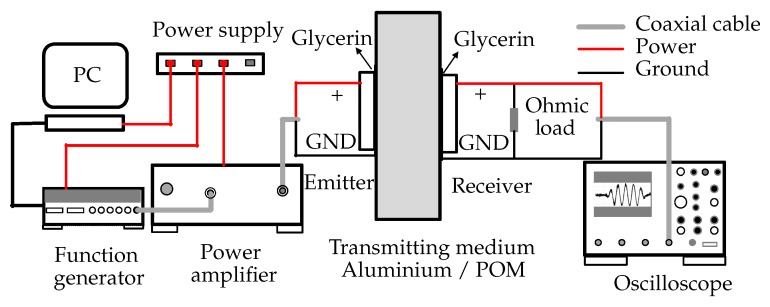
Experimental setup for ultrasonic power transmission (UPT).

The experimental setup is shown in Figure 6. The emitter was powered by a Vectawave power amplifier VBA230-35 (Vectawave Technology Ltd, Newport, UK). The wave burst input to the amplifier was generated using a LeCroy Aabstudio 1104 (Lecroy Corp., Chestnut Ridge, NY, USA), and managed through a virtual instrumentation environment developed in LabVIEW (LabVIEW 2014, National Instruments, Austin, TX, USA).

On the other side of the wall, the receiver was connected with pure ohmic loads with a breadboard, and we measured the voltage across the ohmic load using a digital oscilloscope LeCroy Waverunner 64MXi (Lecroy Corp., Chestnut Ridge, NY, USA) connected through coaxial cables. The parasitic effects of the breadboard and coaxial cable were measured with an impedance analyzer and included in the KLM model. The transducers were clamped to the medium wall with Glycerin as a coupling layer to avoid any air gap. This fixation method allowed decoupling and reusing the transducers several times. Although not optimal, we could achieve reasonable efficiency in power transmission.

### 5.1. Results: Optimal Electric Loads to Maximize Power Transfer

We performed frequency sweeps between 0.6 MHz and 1.1 MHz with 10 MHz increments of the emitter, with ohmic loads in steps of 22 Ω attached to the receiver. As mentioned before, to avoid standing waves in the medium due to back reflection, we performed frequency sweeps with wave bursts. We used a burst of five cycles for aluminium and ten cycles for POM, based on the wavelength and thickness of the wall.

**Figure 7:**
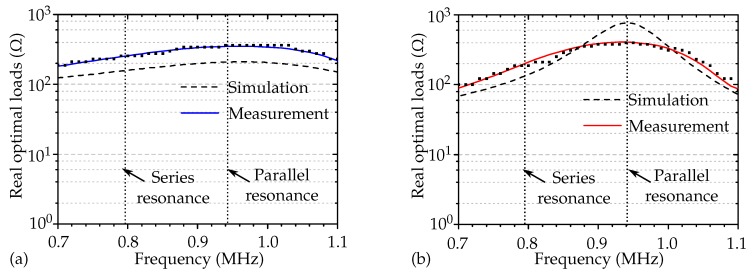
Simulated and measured real optimal electric loads at the receiver to maximize power transfer for each analyzed driving frequency with (**a**) aluminium as the transmitting medium and (**b**) POM as the transmitting medium. The squares show the measured data points, and the solid line is obtained by filtering discrete data points.

Figure 7a,b show the simulated and measured optimal ohmic loads for both aluminium and POM as the transmitting medium. The squares in the plot show the measured data points from the frequency sweep for each analyzed load. The discrete data points were smoothed using a Savitzky–Golay filter [31] to examine the deviation from the predicted optimal electric loads. The solid line in the plot shows the filtered data.

The simulated load magnitudes differed slightly from the measurements. This was due to the inaccuracy caused by the backing medium. In our experimental setup, the transducers were pressed from the backside to the transmitting medium, leading to a not well-defined back acoustic boundary condition. In Figure 7b, we do not see a clear peak in the measured ohmic loads at parallel resonance, since the piezoelectric transducer was under stress, and that caused damping. Furthermore, the KLM model was one-dimensional, and the influence of shear waves could cause inaccuracies in the optimal load prediction.

### 5.2. Results: Power Transfer Frequency

**Figure 8:**
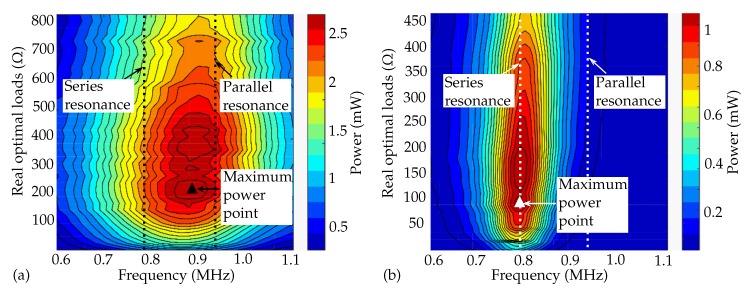
Measured power with real electric loads at the receiver with (**a**) aluminium as the transmitting medium and (**b**) POM as the transmitting medium.

We then measured the power delivered to the attached ohmic loads at the receiver through the frequency sweeps for aluminium and POM as the transmitting medium, shown in Figure 8a,b. To identify the optimal operational frequency of an acoustic power link, with two piezoelectric transducers used to transmit and receive ultrasonic pulses, we evaluated the two-way voltage transfer function $H_{ER}\left(p\right)$, which is the product of the emission and reception transfer function $H_{E}\left(p\right)$ and $H_{R}\left(p\right)$:

(6) $$H_{ER}\left(p\right)=H_{E}\left(p\right)H_{R}\left(p\right).$$

Compared to the simulation results of the emitter and receiver in Figure 5, due to the low acoustic impedance mismatch between the aluminium (13.7 MRayl) and the 1–3 composite piezoceramic transducer (25.6 MRayl), the emitter showed broadband transmission, and the maximum power point was shifted towards the parallel resonance, as the maximum receiving voltage sensitivity of the receiver occurred at the parallel resonance frequency. On the other hand, for POM, the maximum power still occurred at the series resonance frequency, as shown in Figure 8b, due to narrowband transmission.

## 6. Conclusions

Wireless ultrasonic power transfer offers an excellent alternative whenever the location or the size restricts the usage of a battery or inductive coupling. In this paper, we outlined how to enhance the performance of an acoustic power link, based on the properties of the piezoelectric transducer, attached optimal electric load, and acoustic properties of the medium. Transmission of electric energy through an aluminium and a POM wall was achieved indirectly through thickness-stretch vibrations of the wall together with two piezoelectric transducers working in TE mode, one operated as the emitter and the other as the receiver. The frequency response characteristics of the ultrasonic transducers were studied with the KLM model. Interestingly, the output power at the receiver side did not necessarily peak at the fundamental resonant frequency. In contrast, the operating frequency for maximum power transfer depended on the impedance mismatch between the medium and PZT. We evaluated the power maximization approach to calculate the optimal electric load with the KLM model, and the predictions were similar to the measurements.

In this work, we identified the key parameters that influenced the performance of a UPT system. In future work, we will validate our model further with more material media such as steel and different coupling methods. We also aim to find the thermal influences for high power operation and high temperature UPT.

## Figures and Tables

**Table 1:** The 1–3 composite transducer extracted properties.

Parameters	Before Mounting	After Mounting
Thickness (mm)	2.10	2.10
Radius (mm)	7.98	7.98
Density (kgm^-3^)	6470	6470
Impedance (MRayl)	25.6	25.6
Series Resonance (kHz)	792 + 20j	772 + 51j
Parallel Resonance (kHz)	945 + 11j	924 + 45j
Stiffness (kNmm^-2^)	102 + 2.4j	97 + 9.9j
Permittivity	568 − 17.5j	575 − 7.6j
Coupling factor	0.58 − 0.008j	0.57 − 0.013j
